# Association of midlife antibiotic use with subsequent cognitive function in women

**DOI:** 10.1371/journal.pone.0264649

**Published:** 2022-03-23

**Authors:** Raaj S. Mehta, Paul Lochhead, Yiqing Wang, Wenjie Ma, Long H. Nguyen, Bharati Kochar, Curtis Huttenhower, Francine Grodstein, Andrew T. Chan

**Affiliations:** 1 Clinical and Translational Epidemiology Unit, Massachusetts General Hospital and Harvard Medical School, Boston, Massachusetts, United States of America; 2 Department of Biostatistics, Harvard T.H. Chan School of Public Health, Boston, Massachusetts, United States of America; 3 Rush Alzheimer’s Disease Center, Rush University Medical Center and Department of Internal Medicine, Rush Medical College, Chicago, Illinois, United States of America; 4 Channing Division of Network Medicine, Brigham and Women’s Hospital and Harvard Medical School, Boston, Massachusetts, United States of America; Texas A&M University College Station, UNITED STATES

## Abstract

The gut microbiome is increasingly recognized to play a role in cognition and dementia. Antibiotic use impacts the gut microbiome and has been linked with chronic disease. Despite these data, there is no evidence supporting an association between long-term antibiotic use in adults and cognitive function. We conducted a prospective population-based cohort study among 14,542 participants in the Nurses’ Health Study II who completed a self-administered computerized neuropsychological test battery between 2014–2018. Multivariate linear regression models were used to assess if chronic antibiotic use in midlife was associated with cognitive impairment assessed later in life. Women who reported at least 2 months of antibiotic exposure in midlife (mean age 54.7, SD 4.6) had lower mean cognitive scores seven years later, after adjustment for age and educational attainment of the spouse and parent, with a mean difference of -0.11 standard units for the global composite score (P_trend_ <0.0001), -0.13 for a composite score of psychomotor speed and attention (P_trend_ <0.0001), and -0.10 for a composite score of learning and working memory (P_trend_ <0.0001) compared with non-antibiotic users. These differences were not materially changed after multivariate adjustment for additional risk factors, including comorbid conditions. As a benchmark, the mean difference in score associated with each additional year of age was (-0.03) for global cognition, (-0.04) for psychomotor speed and attention, and (-0.03) for learning and working memory; thus the relation of antibiotic use to cognition was roughly equivalent to that found for three to four years of aging. Long-term antibiotic use in midlife is associated with small decreases in cognition assessed seven years later. These data underscore the importance of antibiotic stewardship, especially among aging populations.

## Introduction

The brain-gut axis [1] as mediated by the intestinal microbiome may contribute to neuropsychiatric illnesses including depression [2], schizophrenia [3], autism [4], and anxiety [5]. Emerging evidence also suggests a role of the gut microbiome in the etiopathogenesis of dementia. Animal data indicate that alterations in oral [6] and intestinal bacteria [7,8] may be involved in formation of amyloid plaques. Small, cross-sectional studies suggest large taxonomic differences between participants with and without Alzheimer’s Disease [9].

Chronic antibiotic use has been associated with an increased risk of conditions related to chronic inflammation, including obesity [10], cancer [11], and colonic neoplasia [12]. One recent study found that antibiotic use in the fifth and sixth decades of life–but not at older or younger ages–was associated with incident cardiovascular disease [13]. These associations may be mediated by antibiotic-induced alterations in gut microbial communities [14,15]. Despite recovery of some bacterial species after completion of antibiotic treatment, overall shifts in gut microbial taxonomic communities and changes to certain bacterial genes persist months to years after drug exposure [16–18].

Evidence linking antibiotic use with cognition is limited. Experimental studies have shown that antibiotic administration causes changes in behavior [19] and impaired spatial memory [20]. In humans, early life antibiotic exposure is associated with worse cognitive outcomes in children [21,22], and among patients with Alzheimer’s disease, twelve months of treatment with doxycycline and rifampin compared to placebo led to declines in cognition [23]. To our knowledge, there are no studies examining midlife antibiotic use with subsequent cognitive function in participants without dementia.

Therefore, we investigated the association between duration of midlife antibiotic use (within the sixth decade of life) with validated measures of cognition assessed a mean of 7.0 (SD 0.7) years later among women enrolled in the Nurses’ Health Study II (NHS2) where information on cumulative antibiotic use during adulthood, as well as other lifestyle risk factors, has been prospectively collected.

## Materials and methods

### Study population

The Nurses’ Health Study II is an ongoing US-nationwide prospective cohort study, which began in 1989 with the enrollment of 116,430 female nurses aged 25–42 years [24]. Every two years, participants return questionnaires with detailed information on lifestyle, medication, and health-related factors. Follow-up on each biennial questionnaire exceeds 90%. The Human Research Committees of the Brigham and Women’s Hospital approved this study. Consent was implied by return of written questionnaires.

### Assessment of midlife antibiotic use

In 2009, NHS2 participants (mean age 54.7, SD 4.6) were asked to report their total duration of antibiotic use in 7 categories (ranging from none to 3+ years) over the preceding 4 years, which was then called midlife antibiotic use. Participants were asked to report the most common reason that an antibiotic was used with the following response categories: respiratory infection, urinary tract infection (UTI), acne/rosacea, chronic bronchitis, dental, and other reason.

### Assessment of cognition

A mean of 7.0 years (SD 0.7) after our primary exposure was ascertained from the 2009 questionnaire, we administered CogState, a standardized and validated, self-administered, online cognitive battery in which deficits have previously been shown to be associated with dementia [25]. Between 2014 and 2018, 15,129 women completed the neuropsychological battery using a computer at home. The characteristics of women who accepted the CogState invitation were similar to those who did not respond [26]. The battery comprised four tasks presented in the following order: *Detection*, measuring psychomotor function and information processing speed, in which women press a key when a playing card on the screen flips over; *Identification*, measuring vigilance and visual attention, in which women press a key when a red card flips over; *One Card Learning*, which measures visual learning and short-term memory, in which women are shown playing cards and asked to remember if they have seen the card previously; and *One Back*, which measures attention and working memory, in which women are asked if the card on the screen is identical to the card shown just before [26,27]. As per prior studies [26,27], we generated three composite scores by averaging standardized scores (z-scores) from individual CogState tasks. A composite score of psychomotor speed and attention was derived from Detection and Identification, a composite measure of learning and working memory from One Card Learning and One Back, and a composite score for global cognition from the z-scores for all four tasks.

### Assessment of other potential confounding variables

As described in prior studies [27], as a proxy for socioeconomic status, the nurse’s spouse’s highest educational level was queried in 1999 (high school, undergraduate, postgraduate, unmarried/unknown). Participants were asked about the highest educational level of either parent in 2005 (high school, undergraduate, postgraduate, unknown).

Diet quality derived from food frequency questionnaires was assessed using the Alternative Health Eating Index (AHEI) 2010 without alcohol, returned every 4 years [28]. As in prior studies [27], we used cumulative average values from 1999–2011 to minimize variance and represent long-term patterns. Regular use of antidepressants and symptoms of depression was assessed in 2013. Along with dietary data collection, alcohol consumption was assessed according to number of drinks per day (beer, wine, liquor) every 4 years, and then converted to grams of alcohol per day: “none”, “1–14 g/day”, or ≥15 g/day.

The remainder of covariates, including body mass index, smoking status, regular use of multivitamins, history of high blood pressure (yes/no), history of high cholesterol (yes/no), history of type 2 diabetes (yes/no), history of emphysema (yes/no), history of stroke (yes/no), history of myocardial infarction (yes/no) regular use of aspirin or nonsteroidal anti-inflammatory drugs (yes/no), physical activity (continuous, MET-hours/week) were assessed at the time of exposure in 2009.

### Statistical analysis

Among the 15,129 women who completed the CogState battery, consistent with previous studies [27] we excluded participants who failed integrity checks on all 4 CogState tasks, who had insufficient data to calculate at least 1 composite cognitive score, or did not provide antibiotic data, leaving 14,542 women for analysis. For the primary analysis, we examined midlife antibiotic use (categorized as none, 1–14 days, 15 days– 2 months, and 2+ months) in relation to the following three composite scores: psychomotor speed and attention, learning and working memory, and global cognition, using linear regression models. Our base model was adjusted for age at the time of cognitive assessment and educational attainment of parents and spouse. Our multivariable models were additionally adjusted for body mass index, regular use of antidepressants or depression symptoms, smoking status, regular use of multivitamins, high blood pressure, high cholesterol, type 2 diabetes, emphysema, history of stroke, history of myocardial infarction, regular use of aspirin or nonsteroidal anti-inflammatory drugs, physical activity, and dietary scores. Relative to the referent category of those who used no antibiotics (none), we computed β estimates (mean differences) and 95% confidence intervals (CI) for cognitive scores for each category of use duration as per prior studies [27]. To test for linear trend, we used the median of each category of duration of use as a continuous variable. In secondary analyses, we also examined the association between the reason for midlife antibiotic use with cognitive scores. To address confounding by indication and/or effect modification by age, chronic medical conditions, or other medications, we performed stratified analyses by presence of comorbidities (history of type 2 diabetes, myocardial infarction, stroke, and emphysema), age > 65, and regular use of antidepressants. Analyses were performed using SAS v 9.4 (SAS Institute, Cary, NC).

P < 0.05 was considered significant.

## Results

We identified 14,542 women in the NHS2 cohort who completed cognitive testing and completed medication questionnaires. The mean age at the time of cognitive testing between 2014–2018 was 61.7 years (SD 4.6). Women who reported a longer duration of antibiotic use at midlife (mean age 54.7, SD 4.6) were generally similar to women who did not report any chronic antibiotic use in terms of alcohol intake, educational attainment of parents and spouse, and BMI; however, they were more likely to use antidepressants and aspirin and to have a history of myocardial infarction, stroke, and emphysema (**Table 1**).

**Table 1:** Age-standardized characteristics of NHS2 participants with cognitive assessments (2014–2018), according to midlife antibiotic use reported between 2005 and 2009.

	None (n = 3,398)	< 15 d (n = 5,816)	15 d– 2 mo (n = 4,133)	2 mo+ (n = 1,195)
Mean age at cognitive assessment, y (SD)^a^	61.7 (4.6)	61.7 (4.6)	61.8 (4.7)	61.5 (4.6)
Spouse’s education level, %				
High school or less, %	14.4	15.4	15.2	16.5
College degree, %	42.5	43.0	44.0	38.2
Graduate school, %	31.2	30.2	28.3	29.5
Unmarried/unknown, %	11.9	11.4	12.5	15.8
Highest parental education level, %				
High school or less, %	45.3	48.7	48.1	47.3
College degree, %	25.4	23.9	23.9	24.9
Graduate school, %	27.0	24.1	24.8	24.5
BMI, kg/m2 (SD)	26.2 (5.7)	27.2 (6.1)	28.1 (6.5)	28.7 (6.9)
Physical activity, MET-h/wk (SD)	24.7 (22)	23.7 (22.2)	21.3 (20.6)	21 (21.3)
Smoking status, %				
Never	68.2	66.2	64.0	64.3
Former	28.8	29.1	31.0	29.5
Current	2.9	4.6	4.8	6.1
Alcohol consumption, %				
None, %	14.5	12.6	12.3	11.4
1–14 g/day, %	63.1	64.6	62.3	61.6
Type 2 diabetes, %	3.3	4.3	6.6	9.8
Myocardial infarction, %	0.6	1.2	1.0	1.4
Hypertension, %	25.5	31.6	35.3	41.0
High cholesterol, %	44.8	50.2	55.2	59.2
Stroke, %	0.6	1.0	1.2	1.5
Emphysema, %	0.8	1.8	4.1	7.1
Regular anti-depressant use, %^b^, ^c^	14.1	19.6	25.7	32.3
Regular aspirin use, %^b^	7.6	10.3	10.3	10.9
Regular NSAID use, % b	35.7	40.8	44.0	44.1
Regular multivitamin use, %^b^	52.2	54.1	56.6	52.7
AHEI 2010^d^	54.6 (10.2)	53.8 (10.2)	52.9 (10)	52.6 (9.8)

^a^Value is not age-adjusted.

^b^Participants who responded ‘yes’ when asked whether they had regularly used aspirin, NSAIDs, antidepressants, or multivitamins over the preceding two years.

^c^Antidepressant use assessed at time of CogState testing.

^d^Alternative Healthy Eating Index, higher scores associated with lower risks of chronic diseases.

Increasing total exposure to antibiotics in midlife was significantly associated with poorer scores for all three cognitive domains (**Table 2**). Compared to non-users, women who used antibiotics for at least two months had mean scores that were lower by 0.11 standard units for global cognition (P_trend_ = 0.002), 0.13 for psychomotor speed and attention (P_trend_ = 0.004), and 0.10 for learning and working memory (P_trend_ = 0.03), after adjustment for age and educational attainment of the parent and spouse. This association remained after multivariable adjustment for risk factors for cognitive decline, including medical comorbidities. To assist in interpretation of the CogState score data, we found the mean score differences associated with each additional year of age to be -0.03 for global cognition, -0.04 for psychomotor speed and attention, and -0.03 for learning and working memory (**Table 2**). Thus, the effect estimates that we found for antibiotic use were equivalent to the effect estimates we would expect for three to four years of aging.

**Table 2:** Mean differences in cognitive scores according to midlife antibiotic use.

Composite score	Estimates (95% CI) for mean difference in cognitive scores according to duration of midlife antibiotic use**				
	None	< 15 d	15 d– 2 mo	2 mo+	P_trend_*
**Global cognition**					
Model 1^†^	0.00	-0.02 (-0.05, 0.004)	-0.04 (-0.07–0.007)	-0.11 (-0.16,-0.07)	<0.0001
Model 2^‡^	0.00	-0.01 (-0.04, 0.01)	-0.02 (-0.05, 0.01)	-0.08 (-0.12,-0.03)	0.002
**Psychomotor speed, attention**					
Model 1^†^	0.00	-0.005 (-0.04, 0.03)	-0.02 (-0.06, 0.02)	-0.13 (-0.19,-0.07)	<0.0001
Model 2^‡^	0.00	0.002 (-0.04, 0.04)	-0.002 (-0.04, 0.04)	-0.10 (-0.16,-0.04)	0.004
**Learning, working memory**					
Model 1^†^	0.00	-0.04 (-0.07,-0.01)	-0.06 (-0.09,-0.02)	-0.10 (-0.14,-0.05)	<0.0001
Model 2^‡^	0.00	-0.03 (-0.06,0.00)	-0.03 (-0.07,0.00)	-0.06 (-0.11,-0.01)	0.03

* Tests for trend were conducted using the median of the duration of antibiotic therapy as a continuous variable.

^†^Model 1: Adjusted for age and educational attainment of parents and spouse.

^‡^Model 2: Adjusted for variables included in Model 1 plus smoking status, body mass index, alcohol intake, physical activity, antidepressant use and symptoms of depression, aspirin use, NSAID use, Alternative Healthy Eating Index score, multivitamin use, and history of hypertension, stroke, type 2 diabetes, myocardial infarction, emphysema, or high cholesterol. All were assessed at the time of exposure except antidepressant use and symptoms of depression, which were asked about at the time of CogState testing.

**To assist in interpretation of the CogState score data, the mean difference in score associated with one year of age in these models was (-0.03) for global cognition, (-0.04) for psychomotor speed and attention, and (-0.03) for learning and working memory.

The most common indication for chronic antibiotic use was respiratory infections, followed by “other” (including acne), urinary tract infections (UTI), and dental indications. Although sample size was limited within antibiotic use indication subgroups, the most substantial differences in cognitive scores appeared to be in the subgroup of women using antibiotics for respiratory infections or UTIs (global score difference for users vs. non-users was -0.04 and -0.04, respectively) (**S1 Table**).

We examined the association of chronic antibiotic use and global cognition within subgroups stratified by age, presence or absence of history of diabetes mellitus, emphysema, MI, stroke, or COPD; and regular use or non-use of antidepressants. The associations between antibiotic use with cognitive scores were generally similar between strata, and p_int_ were not significant using the continuous variable for trend (**Table 3**).

**Table 3:** Mean differences in global cognitive scores according to midlife antibiotic use within specific subgroups.

Composite score	Estimates (95% CI) for mean difference in cognitive scores**					
	None	< 15 d	15 d– 2 mo	2 mo+	P_trend_*	P_int_
**Global cognition**						
Age ≤ 65 (10,600) (@ time of CogState)						
Model^‡^	0.00	-0.009 (-0.04, 0.02)	-0.02 (-0.06, 0.01)	-0.07 (-0.12, -0.02)	0.006	
Age > 65 (4,182) (@ time of CogState)						0.30
Model^‡^	0.00	-0.03 (-0.08, 0.03)	-0.003 (-0.06, 0.06)	-0.10 (-0.19, -0.008)	0.17	
**Global cognition**						
No use of antidepressants, 2013 (13,417)						
Model^‡^	0.00	-0.01 (-0.04, 0.02)	-0.01 (-0.05, 0.02)	-0.09 (-0.14, -0.03)	0.007	
Use of antidepressants, 2013 (n = 3,116)						0.76
Model^‡^	0.00	-0.007 (-0.08, 0.06)	-0.02 (-0.09, 0.05)	-0.06 (-0.15, 0.03)	0.13	
**Global cognition**						
Hx of MI/CVA/emphysema/DM2 (n = 1,365)						
Model^‡^	0.00	-0.02 (-0.15, 0.10)	-0.06 (-0.19, 0.06)	-0.12 (-0.27, 0.02)	0.06	
None of the above (n = 13,417)						0.80
Model^‡^	0.00	-0.02 (-0.04, 0.01)	-0.02 (-0.05, 0.02)	-0.08 (-0.13, -0.03)	0.005	

* Tests for trend were conducted using the median of the duration of antibiotic therapy as a continuous variable.

‡Model: Adjusted for age and educational attainment of parents and spouse plus smoking status, body mass index, alcohol intake, physical activity, antidepressant use and symptoms of depression, aspirin use, NSAID use, Alternative Healthy Eating Index score, multivitamin use, and history of hypertension, stroke, type 2 diabetes, myocardial infarction, emphysema, or high cholesterol (with exception of the stratification variable). All were assessed at the time of exposure except antidepressant use and symptoms of depression, which were asked about at the time of CogState testing.

** To assist in interpretation of the CogState score data, the estimated difference in score associated with one year of increasing age was -0.03 for overall cognition, -0.04 for psychomotor speed and attention, and -0.03 for learning and working memory.

Abbreviations: Hx, history; MI, myocardial infarction; CVA, cerebrovascular accident; DM2, Type 2 diabetes mellitus.

## Discussion

In a cohort of over 14,000 women, we observed that antibiotic use in midlife was significantly associated with subsequent poorer scores for global cognition, learning and working memory, and psychomotor speed and attention on a cognitive assessment administered a mean of 7 years later. This relationship was associated with longer duration of antibiotic use and persisted after adjustment for many potential confounding factors. We also found consistent associations within strata defined according to medical comorbidities, age, and antidepressant use. To our knowledge, our study represents the first large study of chronic long-term use of antibiotics and subsequent cognition.

Clinical observations have linked extreme neurologic changes such as encephalopathy to short-term antibiotic use. Among hospitalized patients, psychosis may occur within days after fluroquinolone or macrolide administration [29]. Furthermore, rates of delirium caused by cefepime, a fourth-generation cephalosporin approach 15% [30]. Although such effects have been largely attributed to direct central nervous system neurotoxicity, recent experimental studies suggest a more complex antibiotic-cognition relationship. High-dose, broad-spectrum antibiotic administration in mice (designed to mimic germ-free states) caused cognitive deficits, altered gut microbial profiles, and modulated cognition-related signaling molecules like neuropeptide Y, serotonin transporter, and NA subunit [19].

Epidemiologic data for long-term use of antibiotics in relation to cognition are limited and examine much different contexts than our study. Antibiotic exposure in infancy was associated with lower overall cognitive and verbal comprehension abilities at 11 years of age, as well as depressive symptoms at 3 years [21,22]. These two studies suggest a potential latency between antibiotic use and later neurocognitive symptoms, although it is difficult to extend findings from infant antibiotic use to those in our research of adults. For cardiovascular disease, which is associated with cognition, we previously observed an association between antibiotic use at mid-life (age 40–60) and risk of incident cardiovascular disease with aging (beyond age 60) in our cohort of older women, the Nurses’ Health Study [13]. Finally, in a multicenter randomized clinical trial among patients with Alzheimer’s disease, 12 months of treatment with antibiotics led to declines in cognitive scores compared to placebo [23]. Thus, there is some support for relations of antibiotic use to long-term health in aging.

Given the profound effect of antibiotic use on the gut microbiome—with prior studies showing alterations in functional potential at 2 [14] and 4 years [18] after antibiotic exposure—the gut-brain axis could be a possible mechanism for linking antibiotics to cognitive function. Indeed, over the last decade, there has been emerging data linking intestinal bacteria to the brain-gut axis [1–5]. Recent cross-sectional data from small studies indicate large taxonomic differences in gut microbiomes from patients with Alzheimer’s Disease when compared to healthy controls [9,31]. Experimental data demonstrate potential causal mechanisms that might underly these associations. Inoculation of mice with the oral bacterium *Porphyromonas gingivalis* led to greater production of brain amyloid plaques, the hallmark pathology of Alzheimer dementia [6]. In another study, modulation of gut microbes in mice with an antibiotic cocktail led to reductions in accumulation of brain amyloid tangles [7]. Further still, mice colonized by *Lactobacillus* species performed better on retention tests than uncolonized mice [32].

Strengths of our analysis include our large sample, and extensive characterization of participants, enabling control for multiple potential confounding variables, including socioeconomic status, diet, other medications, and medical illnesses. Furthermore, the validity of CogState has been established previously [33].

We acknowledge several limitations. Our antibiotic data do not contain information about route or specific antibiotic type. Additionally, antibiotic information was based on self-report several years after use, and thus may be subject to misclassification and/or recall bias. Further, our data are limited to women. Since cognitive function may differ in women and men, as well as across racial and ethnic groups, further studies in diverse populations are needed. Additionally, due to a lack of power, we cannot directly link microbial features with cognition scores in this cohort. Finally, as in any observational study, we cannot exclude unmeasured and residual confounding. We acknowledge that in this hypothesis-generating study, participants who used antibiotics were more likely to use other medications and/or have comorbid conditions, which could be a proxy for poorer health status, such as chronic infection, which has been linked to cognition [34]. Nevertheless, the associations we observed persisted after adjustment for and stratification by multiple comorbidities.

## Conclusions

In summary, we found that chronic antibiotic use during midlife was associated with minor decreases in cognitive scores assessed a mean of 7 years later. These data provide a better understanding of potential complications of antibiotics throughout life, as well as generate hypotheses about the role of the gut microbiome in cognition.

## Supporting information

S1 Table Mean differences in cognitive scores according to reason for midlife antibiotic use. (DOCX)

## Abbreviations

Abx: antibiotics
BMI: body mass index
CI: confidence interval
HR: hazard ratio
MGX: metagenomics
NHS II: Nurses’ Health Study II
