# Supplementary material for: Association of midlife antibiotic use with subsequent cognitive function in women
Source: PLoS One. 2022 Mar 23;17(3):e0264649. doi: 10.1371/journal.pone.0264649 (PMC8942267; doi:10.1371/journal.pone.0264649)
Supplement: S1 Table — (DOCX) [file pone.0264649.s001.docx]

**Supporting information**

**S1 Table.** Mean differences in cognitive scores according to reason for midlife antibiotic use.

| **Composite score** | **Estimates (95% CI) for mean difference in cognitive scores according to indication for antibiotic use in midlife** | | | | |
| --- | --- | --- | --- | --- | --- |
|  | **None**  (3,634) | **Respiratory***  (5,664) | **UTI**  (1,659) | **Dental**  (1,125) | **Other****  (2,519) |
| **Global cognition** |  |  |  |  |  |
| Model^‡^ | 0.00 | -0.02  (-0.05, 0.009) | -0.03  (-0.07, 0.009) | -0.002  (-0.05, 0.04) | 0.01  (-0.02, 0.05) |
| **Psychomotor speed, attention** |  |  |  |  |  |
| Model^‡^ | 0.00 | -0.001  (-0.04, 0.04) | -0.02  (-0.07, 0.03) | -0.01  (-0.07, 0.05) | 0.02  (-0.03, 0.06) |
| **Learning, working memory** |  |  |  |  |  |
| Model^‡^ | 0.00 | -0.04  (-0.07, -0.008) | -0.04  (-0.08, 0.00) | 0.006  (-0.04, 0.05) | 0.006  (-0.03, 0.04) |
| *Defined as “respiratory infections” or “chronic bronchitis”  **Includes chronic use for acne  ‡Model: Adjusted for age and educational attainment of parents and spouse plus smoking status, body mass index, alcohol intake, physical activity, antidepressant use and symptoms of depression, aspirin use, NSAID use, Alternative Healthy Eating Index score, multivitamin use, and history of hypertension, stroke, type 2 diabetes, myocardial infarction, emphysema, or high cholesterol (with exception of the stratification variable). All were assessed at the time of exposure except antidepressant use and symptoms of depression, which were asked about at the time of CogState testing. | | | | | |
